# Supplementary material for: Regulatory T cells confer a circadian signature on inflammatory arthritis
Source: Nat Commun. 2020 Apr 3;11:1658. doi: 10.1038/s41467-020-15525-0 (PMC7125185; doi:10.1038/s41467-020-15525-0)
Supplement: Supplementary file 1 — Supplementary Information [file 41467_2020_15525_MOESM1_ESM.pdf]

Regulatory T cells confer a circadian signature to inflammatory arthritis.

Hand *et al*

Supplementary Information

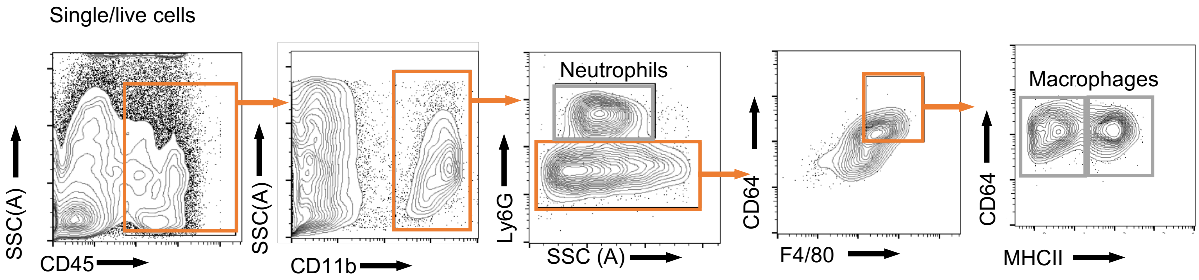

Neutrophils: CD45^{+} CD11b^{+} Ly6G^{+}
Macrophages: CD45^{+} CD11b^{+} Ly6G^{-} F4/80^{+} CD64^{+}

Supplementary Figure 1: **Gating strategy for neutrophils and macrophages from limbs.** Data corresponds to Figures 1b, c, e and f.

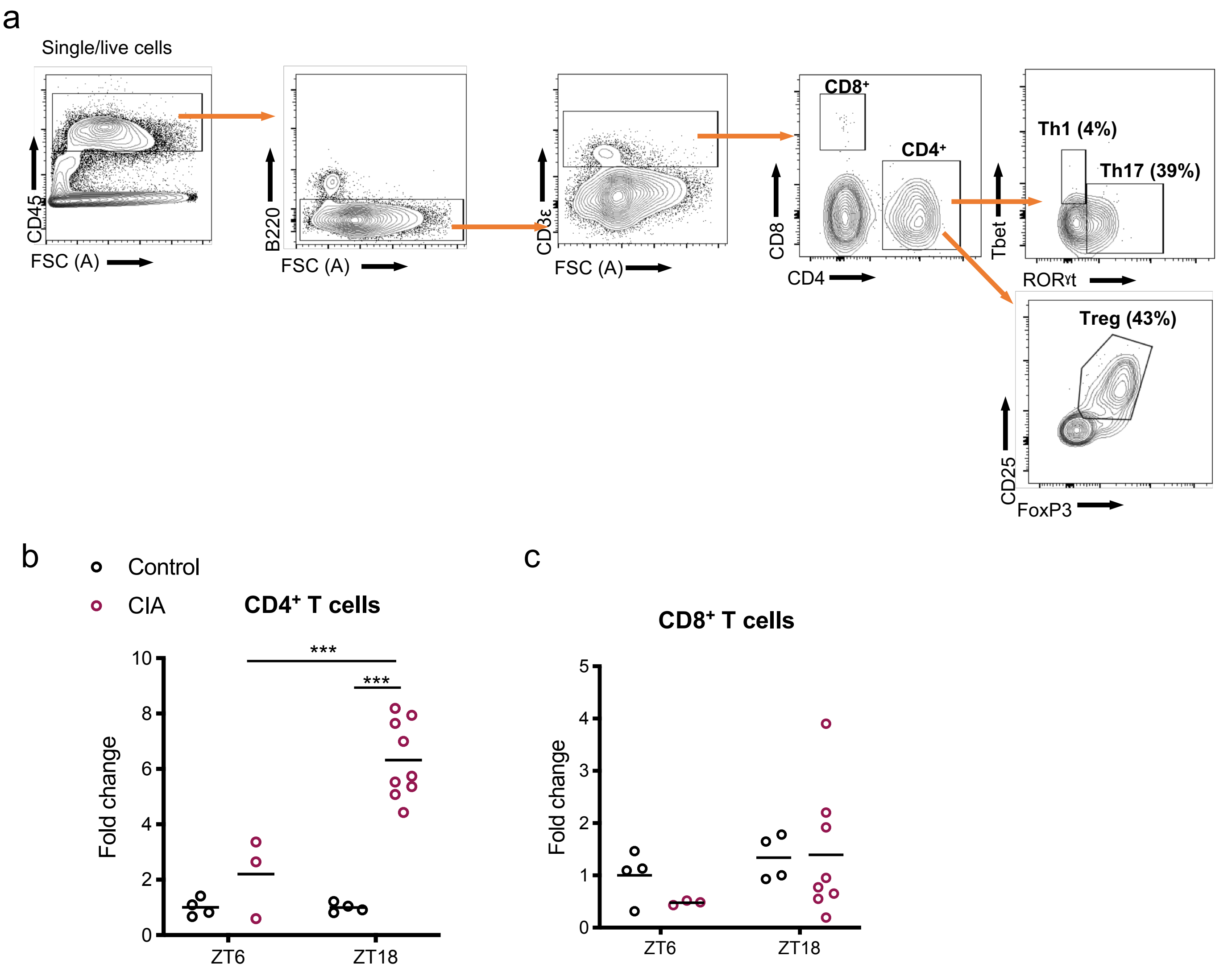

Supplementary Figure 2: T cell subsets in limbs. (a) Flow cytometric gating strategy for T cell subsets, CD8$^+$ and CD4$^+$ Th1, Th17 and Treg, corresponds to Figures 2a-c, 4a,b and d and Supplementary Figure 6a. (b) CD4$^+$ T cells (gating illustrated above) in limbs harvested from control and arthritic mice at ZT6 (Control n=4; CIA n=3) and ZT18 (control n=4; CIA n=9) data normalised to cell numbers in control limbs at ZT6, 2-way ANOVA and Bonferonni post hoc tests. (c) CD8$^+$ T cells (gating illustrated above) in limbs harvested from control and arthritic mice at ZT6 (control n=4; CIA n=3) and ZT18 (control n=4; CIA n=8), data normalised to cell numbers in control limbs at ZT6, 2-way ANOVA and Bonferonni post hoc tests. Graph shows individual data points and mean values. Statistical significance is shown as ***p<0.005. Source data are provided as a Source Data file.

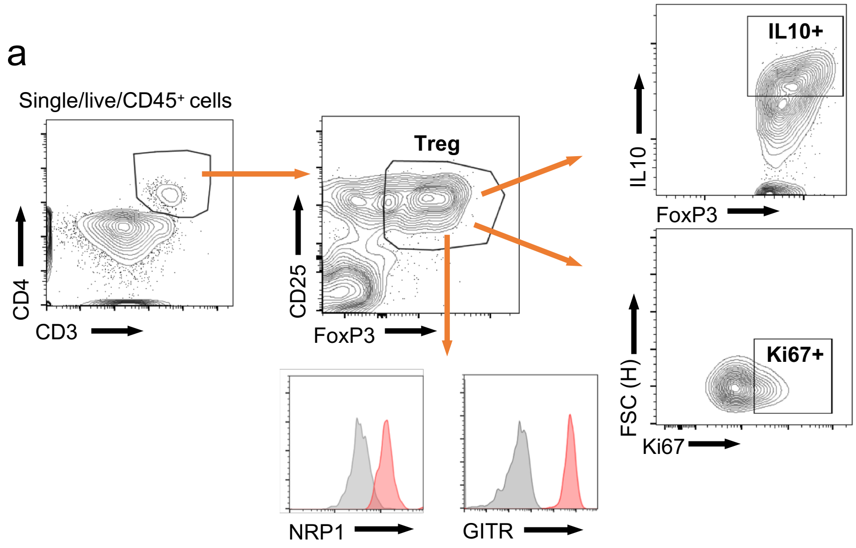

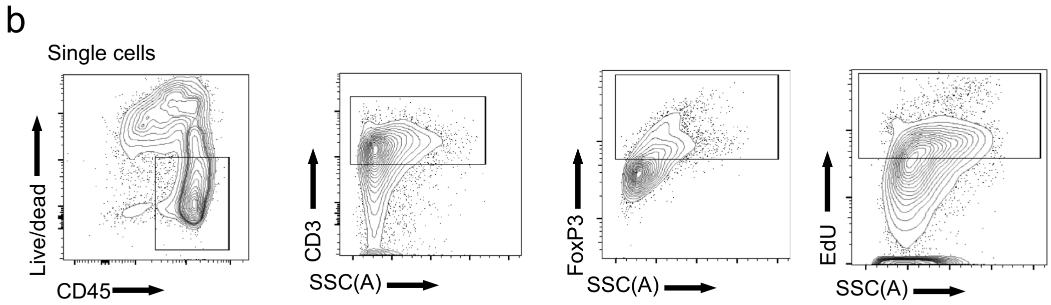

Supplementary Figure 3: Gating strategies for Treg activation and proliferation. (a) Flow cytometric gating strategy for Treg IL10 production and activation/proliferation markers Ki67, NRP1 and GITR. NRP1 and GITR - negative populations are shown in grey, corresponds to Figures 2c-f. (b) Flow cytometric gating strategy for EdU positive Tregs, corresponds to Figure 2d.

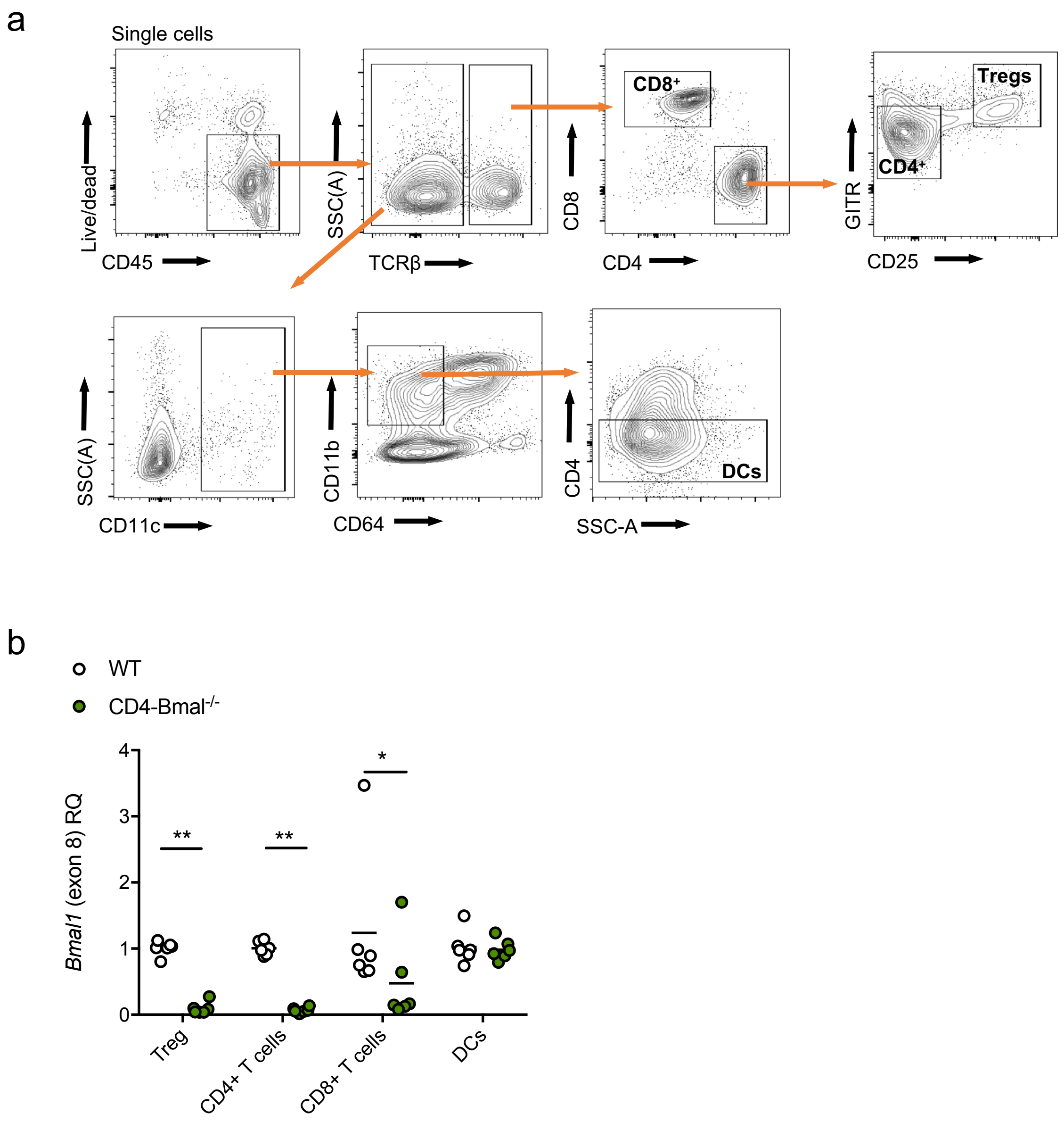

Supplementary Figure 4: **Expression of *Bmal1* (exon 8) in different cell populations.** (a) CD8^{+} T cells (CD45^{+}/TCRβ^{+}/CD8^{+}), CD4^{+} T cells (CD45^{+}/TCRβ^{+}/CD4^{+}/GITR^{-}/CD25^{-}), Tregs (CD45^{+}/TCRβ^{+}/CD4^{+}/GITR^{+}/CD25^{+}) and dendritic cells (DCs: CD45^{+}/TCRβ^{-}/CD11c^{+}/CD11b^{+}/CD64^{-}/CD4^{-}) were sorted at ZT4 from CD4-Bmal1$^{-/-}$ mice and wildtype counterparts. (b) Q-PCR determined expression of *Bmal1 (exon8)* in each cell population (gating illustrated above) (normalised to *gapdh*), samples are made relative to expression in wildtypes, 2-way ANOVA and post hoc Dunnett test, n=6/genotype. Graph shows individual data points and mean values. Statistical significance is shown as *p<0.05 and **p<0.01. Source data are provided as a Source Data file.

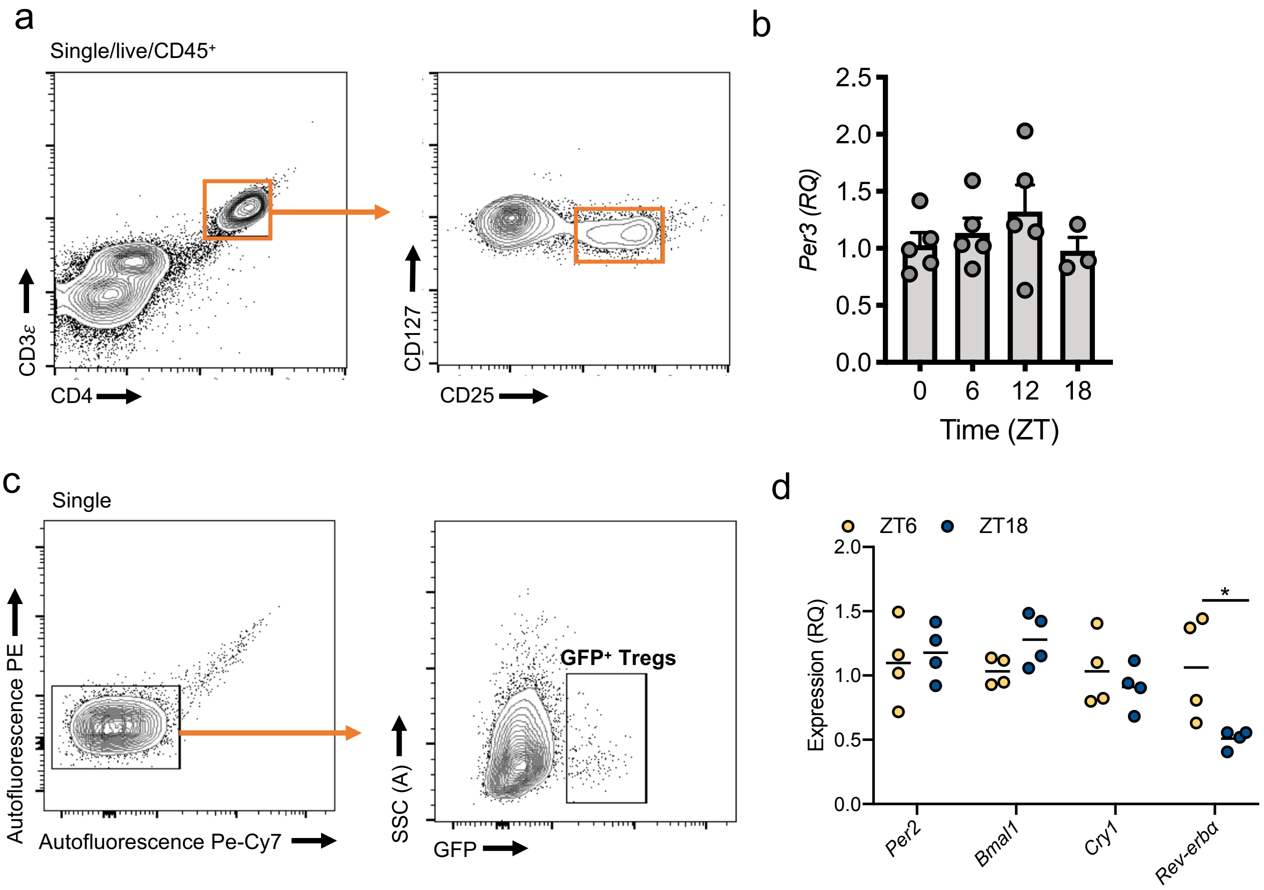

Supplementary Figure 5: Gating strategies for sorting Tregs at different times of the day. (a) Gating strategy for sorting Tregs from lymph nodes using extracellular markers. Single/Live/CD45^{+}/CD3ε^{+}/CD4^{+}/CD127^{-}/CD25^{+} cells were selected, corresponds to Figures 3b-c. (b) Expression of *Per3* in sorted Tregs (gating illustrated above) was quantified by QPCR (normalised to *gapdh*), normalised to ZT0 (ZT0,6,12 n=5; ZT18 n=3) One-way ANOVA and post hoc Bonferonni. Data are presented as mean values ± SEM. (c) GFP^{+} Tregs were sorted from lymph nodes harvested from DEREG mice at ZT6 or ZT18 with no prior antibody staining. (d) QPCR analysis for core clock genes (normalised to β-actin) in GFP^{+} Tregs sorted from lymph nodes (gating illustrated above) at ZT6 and ZT18, n=4/time point, data normalised to ZT0, T tests. Graph shows individual data points and mean values. Statistical significance is shown as *$p<0.05$. Data are provided as a Source Data file.

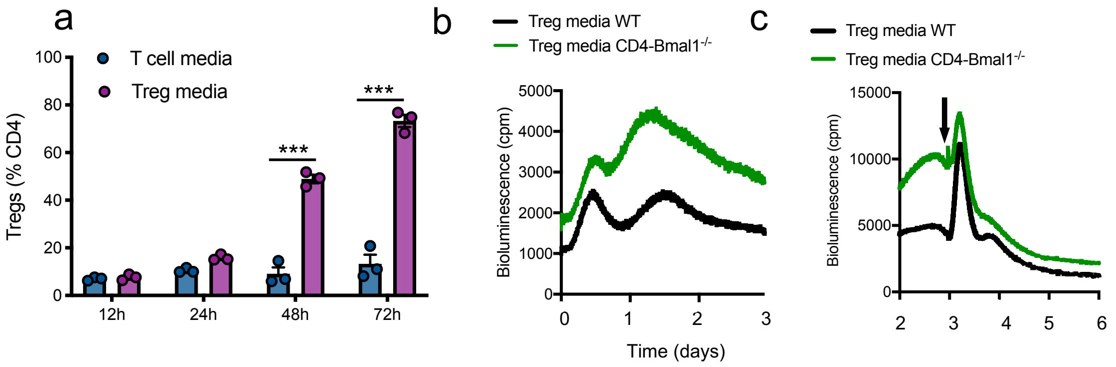

Supplementary Figure 6: PER2 bioluminescence in Tregs from WT and CD4-Bmal1$^{-/-}$ mice. (a) Culturing naïve CD4^{+} T cells in Treg expansion media (IL2, anti-CD28, anti-CD3ε, TGFβ and anti-IFNγ) results in significantly increased Treg numbers by Day 2, n=3/time point, 2-way ANOVA with post-hoc Bonferonni. Data are presented as mean values ± SEM. (b) Naïve CD4^{+} T cells from wildtype and CD4-Bmal1$^{-/-}$ mice on a PER2::luc background show altered bioluminescence in response to Treg expansion media. (c) After the initial response, re-stimulation with anti-CD28 and anti-CD3ε provokes a second PER2::luc response. Statistical significance is shown as ***$p<0.005$. Source data are provided as a Source Data file.

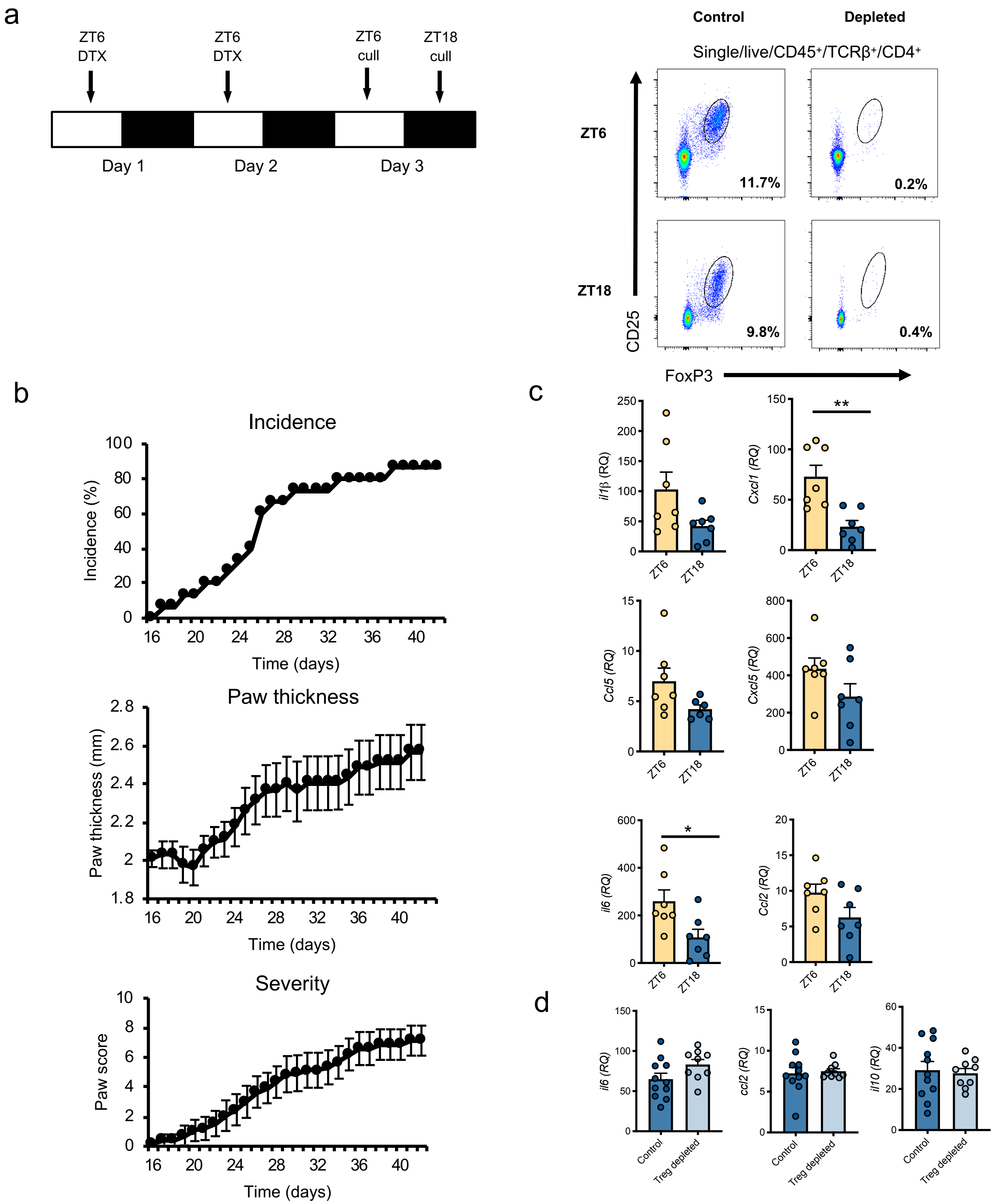

Supplementary Figure 7: Validation of the collagen induced arthritis model in DEREG mice. (a) Depletion of Tregs in the inguinal lymph nodes 24h (ZT6) or 36h (ZT18) after the last dose of diphtheria toxin (DTX), representative of n=3. (b) Susceptibility of DEREG mice to CIA; incidence, hind paw thickness and severity were tracked in a cohort of 15 mice from day 16 post immunization. Data are presented as mean values ± SEM. (c) QPCR analysis of transcripts of inflammatory genes (normalised to *gapdh*) within the joints of arthritic DEREG mice (no DTX treatment) culled at ZT6 (n=7) or ZT18 (n=7). Values were normalised to expression in naïve animals at the relevant time point, T tests. (ZT6 data re-plotted from main Figure 5f). Data are presented as mean values ± SEM. (d) QPCR analysis of transcripts of inflammatory genes (normalised to *gapdh*) within the joints of arthritic DEREG mice culled at ZT18 (control n=11; depleted n=9). Values were normalised to expression in control animals without arthritis, t tests. Data are presented as mean values ± SEM. Statistical significance is shown as *p<0.05 and **p<0.01. Source data are provided as a Source Data file.

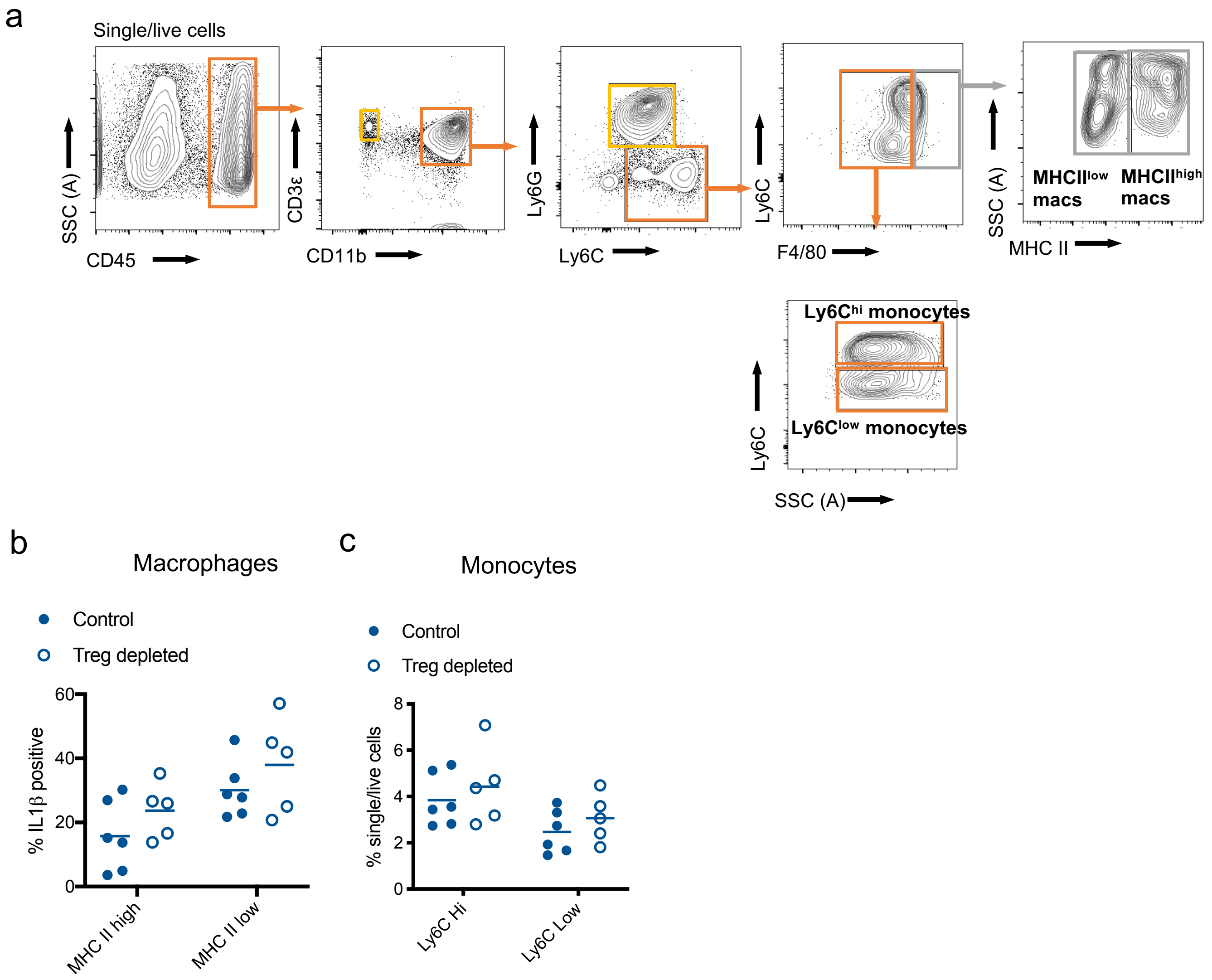

Supplementary Figure 8: Effects of Treg depletion on monocytes and macrophages within inflamed joints. (a) Gating strategy for monocytes and macrophages from inflamed joints, corresponds to Figure 5d and g. (b) Pro-IL1β expression in joint-derived macrophages (gating illustrated above) from CIA DEREG mice, control n=6, depleted n=5. (c) Numbers of different monocyte populations (gating illustrated above) in arthritic joints derived from DEREG CIA mice, control n=6, depleted n=5. Graph shows individual data points and mean values. Source data are provided as a Source Data file.

Supplementary Table 1: Serum cytokines in arthritic DEREG mice. Serum cytokines which were significantly altered in CIA are shown. CIA control n=5, CIA Treg depleted n=6. Comparisons were made with T-tests or Mann Whitney U tests as appropriate, data expressed as mean ± SEM, Statistical significance is shown as *p<0.05.

Cytokine	CIA control (pg/ml)	CIA Treg depleted (pg/ml)	Sig.
IL1α	19.33 ± 2.40	20.36 ± 1.97	
IL1β	31.84 ± 2.67	38.48 ± 5.65	
IL2	17.47 ± 4.58	17.28 ± 2.67	
IL3	12.65 ± 0.70	13.75 ± 2.41	
IL6	59.22 ± 16.02	76.07 ± 12.51	
IL10	108.6 ± 11.99	156.9 ± 49.87	
IL12p40	1329 ± 171.3	2115 ± 188.6	*
IL12p70	278.6 ± 30.89	342 ± 71.69	
IL17	173.4 ± 50.46	175.5 ± 55.07	
G-CSF	1021 ± 155	1423 ± 202.8	
IFNγ	32.24 ± 2.85	39.11 ± 4.76	
CXCL1	117.7 ± 14.24	157 ± 16.71	
CCL2 (MCP1)	283.4 ± 21.92	359.9 ± 25.03	0.0545
CCL3 (MIP1α)	5.51 ± 0.44	6.61 ± 0.54	
CCL4 (MIP1β)	38.6 ± 5.34	42.74 ± 4.92	
CCL5 (Rantes)	264.7 ± 53.43	329 ± 34.4	
TNFα	129 ± 15.9	152.7 ± 28.8	

Supplementary Table 2: Flow cytometry antibodies.

Antibody	Clone	Colours (supplier)	Antibody	Clone	Colours (supplier)
B220	RA3-6B2	APC-Cy7 (Invitrogen)	FoxP3	FJK-16s	BV421 (Invitrogen)<br>FITC (Invitrogen)
CD3ε	145-2C11	PerCP-Cy5.5 (eBioscience)<br>BV711 (Biolegend)<br>PE-Cy7 (Biolegend)<br>PE (Biolegend)<br>Unconjugated (Biolegend)	GITR	DTA-1	APC (Biolegend)
CD4	RM4-5	PE-Texas red (Invitrogen)<br>AF700 (Biolegend)<br>PE (Biolegend)<br>BV711 (Biolegend)	IFNγ	XMG1.2	Unconjugated (Biolegend)
CD8a	53-6.7	Pe-Cy7 (Biolegend)<br>BV785 (Biolegend)	Ki67	SolA15	PE (Invitrogen)
CD11b	M1/70	BV605 (Biolegend)<br>PE (Biolegend)<br>PerCP-Cy5.5 (eBioscience)<br>PeCy7 (Biolegend)	Ly6C	AL-21	AF700 (BD Bioscience)<br>PerCP-Cy5.5 (BD Bioscience)<br>PE-Cy7 (BD Bioscience)
CD11c	N418	BV421 (Biolegend)	Ly6G	1A8	APC-Cy7 (Biolegend)
CD25	PC61.5	AF488 (Invitrogen)<br>PE-Cy7 (Invitrogen)	MHC II	M5/114.15.2	FITC (Biolegend)<br>BV650 (BD Bioscience)
CD28	37.51	Unconjugated (Biolegend)	NRP1	761705	FITC (R&D Systems)
CD45	30-F11	BV650 (Biolegend)<br>AF700 (BD Biosciences)<br>BV510 (Biolegend)	RORγ(t)	B2D	PE (eBioscience)
CD64	X54-5/7.1	FITC (Biolegend)<br>PE (Biolegend)	TBET	4B10	APC (Invitrogen)
CD127	A7R34	PE (Biolegend)	TCRβ	H57-597	BV711 (Biolegend)
CD90.2	30-H12	BV786 (Biolegend)	IL1β pro-form	REA577	Pe-Cy7 (Miltenyi Biotec)
CXCR4	L276F12	PE (Biolegend)<br>Biotin (Biolegend)	IL10	JES5-16E3	BV605 (BD Biosciences)
F4/80	BM8	BV786 (Biolegend)<br>APC (eBioscience)			

Supplementary Table 3: Primer and probe sequences for QPCR analysis. Genes marked with an * were assayed using commercial primer probe sets purchased from ThermoFisher Scientific (product code provided).

Gene	Forward	Reverse	Probe (Fam-Tamra)
β-actin	AGGTCATCACTATTGGCAACGA	CACTTCATGATGGAATTGAATGTAGTT	TGCCACAGGATTCCATACCCAAGAAGG
Bmal1	CGTCGGGACAAAATGAACAG	GAACAGCCATCCTTAGCAC	TACCAACATGCAATGCAATGTCCAGGA A
Ccl2	CTTCTGGGCCTGCTGTTCA	CCAGCCTACTCATTGGGATCA	CTCAGCCAGATGCAGTTAACGCCCC
Ccl5	Mm01302427_m1		
Cry1	CTGGCGTGGAAGTCATCGT	CTGTCCGCCATTGAGTTCTATG	CGCATTTCACATACACTGTATGACCTG GACA
Cxcl1	CTGCACCCAAACCGAAGTC	AGCTTCAGGGTCAAGGCAAG	CACTCAAGAATGGTCGCGAGGC
*Cxcl5**	Mm00436451_g1		
Gapdh	CAATGTGTCCGTCGTCGATCT	GTCCTCAGTGTAGCCCAAGATG	CGTGCCGCCTGGAGAAACCTGCC
Ilβ	TCGCTCAGGGTCACAAAGAAA	CCATCAGAGGCAAGGAGGAA	CATGGCACATTCTGTTCAAAGAGAGCC TG
Il6	CTATACCACTTCACAAGTCGGAGG	TGCACAACTCTTTTCTCATTTCC	TTAATTACACATGTTCTCTGGGAAATCG
*Il10**	Mm01288386_m1		
Per2	GCCTTCAGAACTCATGATGACAGA	TTTGTGTGCGTCAGCTTTGG	ACTGCTCACTACTGCAGCCGCTCGT
Per3	Mm00478120_m1		
*Rev-erbα**	Mm00520708_m1		

Supplementary Table 4: **2-way ANOVAs - P values for interaction and main effects.**

Figure	Description		Interaction	Time of day effect	Disease effect
1b	MHC II^{low} mac		NS	NS	*** (P<0.0001)
	MHC II^{high} mac		NS	NS	*** (P<0.0001)
1c	MHC II^{low} mac	*Per2*	*** (P=0.0006)	*** (P<0.0001)	*** (P=0.0001)
		Cry1	** (P=0.0018)	*** (P<0.0001)	* (P=0.0159)
		Bmal1	* (P=0.0204)	NS	*** (P=0.0001)
		Rev-erb α	*** (P<0.0001)	*** (P<0.0001)	*** (P<0.0001)
	MHC II^{high} mac	*Per2*	** (P=0.0040)	*** (P=0.0005)	*** (P<0.0001)
		Cry1	*(P=0.0254)	*** (P=0.0002)	* (P=0.0216)
		Bmal1	*** (P=0.0002)	** (P=0.0052)	*** (P<0.0001)
		Rev-erb α	*** (P<0.0001)	*** (P<0.0001)	*** (P<0.0001)
1e	Neutrophils		NS	NS	*** (P<0.0001)
1f	Neutrophils	*Per2*	NS	* (P=0.0390)	NS
		Cry1	NS	** (P=0.0094)	NS
		Bmal1	NS	NS	** (P=0.0081)
		Rev-erb α	*** (P<0.0001)	*** (P<0.0001)	*** (P<0.0001)
2a	CD3ε		*** (P=0.0004)	***(P=0.0002)	*** (P<0.0001)
2b	T_h1		NS	NS	* (P=0.0161)
	T_h17		NS	NS	*** (P<0.0001)
2c	Treg		*** (P<0.0001)	*** (P<0.0001)	*** (P<0.0001)
2d	Ki67		NS	*** (P<0.0007)	NS
2e	NRP1		* (P=0.0285)	NS	NS
	GITR		NS	NS	** (P=0.0077)
Figure	Description		Interaction	Time of day effect	Genotype effect
3b	Treg	*Per2*	NS	NS	NS
		Bmal1	NS	NS	*** (P<0.0001)
		Cry1	NS	NS	*** (P<0.0001)
		Rev-erb α	* (P=0.0166)	** (P=0.0014)	*** (P<0.0001)
4b	Treg CXCR4	ILN	NS	*** (P<0.0001)	NS
		Spleen	NS	*** (P<0.0001)	NS
Figure	Description		Interaction	Time of day effect	Disease effect
4e	Corticosterone		** (P=0.0058)	*** (P<0.0001)	NS
Figure	Description		Interaction	Depletion effect	Cell type effect
5g	IL1β		NS	* (P=0.0117)	*** (P<0.0001)
Figure	Description		Interaction	Time of day effect	Disease effect
S2b	CD4+ T cells		** (P=0.0015)	** (P=0.0016)	*** (P<0.0001)
S2c	CD8+ T cells		NS	NS	NS
Figure	Description		Interaction	Depletion effect	Cell type effect
S8b	Macrophage	IL1β	NS	NS	** (P=0.0071)
	Monocytes	number	NS	NS	* (P=0.0169)
