# Supplementary material for: Regulatory T cells confer a circadian signature on inflammatory arthritis
Source: Nat Commun. 2020 Apr 3;11:1658. doi: 10.1038/s41467-020-15525-0 (PMC7125185; doi:10.1038/s41467-020-15525-0)
Supplement: Supplementary file 3 — Reporting Summary [file 41467_2020_15525_MOESM3_ESM.pdf]

nature research

Corresponding author(s): Julie Gibbs
Last updated by author(s): 25/02/2020

# Reporting Summary

Nature Research wishes to improve the reproducibility of the work that we publish. This form provides structure for consistency and transparency in reporting. For further information on Nature Research policies, see Authors & Referees and the Editorial Policy Checklist .

## Statistics

For all statistical analyses, confirm that the following items are present in the figure legend, table legend, main text, or Methods section.

| n/a | Confirmed | |
| --- | --- | --- |
| ☐ | ☒ | The exact sample size (*n*) for each experimental group/condition, given as a discrete number and unit of measurement |
| ☒ | ☐ | A statement on whether measurements were taken from distinct samples or whether the same sample was measured repeatedly |
| ☐ | ☒ | The statistical test(s) used AND whether they are one- or two-sided<br>*Only common tests should be described solely by name; describe more complex techniques in the Methods section.* |
| ☒ | ☐ | A description of all covariates tested |
| ☐ | ☒ | A description of any assumptions or corrections, such as tests of normality and adjustment for multiple comparisons |
| ☐ | ☒ | A full description of the statistical parameters including central tendency (e.g. means) or other basic estimates (e.g. regression coefficient) AND variation (e.g. standard deviation) or associated estimates of uncertainty (e.g. confidence intervals) |
| ☐ | ☒ | For null hypothesis testing, the test statistic (e.g. *F*, *t*, *r*) with confidence intervals, effect sizes, degrees of freedom and *P* value noted<br>*Give P values as exact values whenever suitable.* |
| ☒ | ☐ | For Bayesian analysis, information on the choice of priors and Markov chain Monte Carlo settings |
| ☒ | ☐ | For hierarchical and complex designs, identification of the appropriate level for tests and full reporting of outcomes |
| ☒ | ☐ | Estimates of effect sizes (e.g. Cohen's *d*, Pearson's *r*), indicating how they were calculated |

*Our web collection on statistics for biologists contains articles on many of the points above.*

## Software and code

Policy information about availability of computer code

| | |
| --- | --- |
| Data collection | StepOne software v2.1<br>BioRad microplate manager v2.1<br>Bioplex Manager 6.2<br>FACS DIVAv8.0.1 |
| Data analysis | GraphPad Prism Versions 7.0a and 8.3.1<br>Flow Jo Version 10.5.3<br>Biodare2 (biodare2.ed.ac.uk) |

For manuscripts utilizing custom algorithms or software that are central to the research but not yet described in published literature, software must be made available to editors/reviewers. We strongly encourage code deposition in a community repository (e.g. GitHub). See the Nature Research guidelines for submitting code & software for further information.

## Data

Policy information about availability of data

All manuscripts must include a data availability statement. This statement should provide the following information, where applicable:

- Accession codes, unique identifiers, or web links for publicly available datasets
- A list of figures that have associated raw data
- A description of any restrictions on data availability

The authors declare that the data supporting the findings of this study presented in Figures 1-5 and Supplementary Figures 1-8 are available within the paper and its supplementary information.  Data from photonmultiplier and bioplex experiments are available from the corresponding author upon reasonable request.

# Field-specific reporting

Please select the one below that is the best fit for your research. If you are not sure, read the appropriate sections before making your selection.

[x] Life sciences  [ ] Behavioural & social sciences  [ ] Ecological, evolutionary & environmental sciences

For a reference copy of the document with all sections, see nature.com/documents/nr-reporting-summary-flat.pdf

# Life sciences study design

All studies must disclose on these points even when the disclosure is negative.

**Sample size**

Experiments were designed to ensure the minimum number of animals were used in order to obtain biologically significant results. Determination of experimental and control group sizes were based on previous experience with the models and power analyses of previous results. Animal numbers were based on coefficient variance measures determined in control groups of previous studies. N number calculations were based on achieving 80% power, with 5% type 1 error.

Previous studies used for these calculations include:

Hand et al. (2019) The circadian regulator Bmal1 in joint mesenchymal cells regulates both joint development and inflammatory arthritis. Arthritis Res Ther 21:5

Pariollaud et al (2018) Circadian clock component REV-ERB alpha controls homeostatic regulation of pulmonary inflammation. J Clin Invest 128:2281

Hand et al (2016) The circadian clock regulates inflammatory arthritis. FASEB J 30:3759

Gibbs et al (2014) An epithelial circadian clock controls pulmonary inflammation and glucocorticoid action. Nat Med 20:919

**Data exclusions**

For QPCR data analysis, in order to exclude any erroneous data points generated by experimental error, outliers which deviated markedly from other observations in the group were discovered using the ROUT method in Prism (a method to detect outliers while fitting a curve with a non-linear regression) and were excluded from further analysis.

**Replication**

All in vitro studies were successfully repeated on three separate occasions. In vivo experiments were successfully replicated in at least two independent experiments performed under identical conditions.

**Randomization**

Animals were randomly assigned into experimental groups (e.g. time points for tissue harvest). For the T cell ex vivo studies, wells of cells were allocated randomly to treatment groups (e.g. T cell media or Treg media) and to treatment times.

**Blinding**

Wherever possible investigators were blinded to group allocation during sample collection and analysis. It was not possible to blind investigators to group allocation during tissue collection when experimental groups were defined by time-of-day. However investigators were blind to genotype where applicable. Additionally, for safety reasons it was not possible to blind investigators to treatment group when diphtheria toxin was administered to DEREG mice. Sample analysis was performed with the investigator blind to genotype, time of day and treatment of samples.

# Reporting for specific materials, systems and methods

We require information from authors about some types of materials, experimental systems and methods used in many studies. Here, indicate whether each material, system or method listed is relevant to your study. If you are not sure if a list item applies to your research, read the appropriate section before selecting a response.

## Materials & experimental systems

| n/a | Involved in the study | |
|---|---|---|
| [ ] | [x] | Antibodies |
| [x] | [ ] | Eukaryotic cell lines |
| [x] | [ ] | Palaeontology |
| [ ] | [x] | Animals and other organisms |
| [x] | [ ] | Human research participants |
| [x] | [ ] | Clinical data |

## Methods

| n/a | Involved in the study | |
|---|---|---|
| [x] | [ ] | ChIP-seq |
| [ ] | [x] | Flow cytometry |
| [x] | [ ] | MRI-based neuroimaging |

## Antibodies

**Antibodies used**

Antibody/ Colour/ supplier/ Catalogue number/ Clone/ Dilution
B220/ APC-Cy7/ Invitrogen/ A15399/ RA3-6B2/ 1:200
CD3ε/unconjugated/Biolegend/100314/145-2C11/1:333
CD3ε/ PerCP-Cy5.5/ eBioscience/ 45-0031-82/ 145-2C11/ 1:200
CD3ε/ BV711/ Biolegend/ 100349/ 145-2C11/ 1:200
CD3ε/ PE-Cy7/ Biolegend/ 100320/ 145-2C11/ 1:200

CD3ε/ PE/ Biolegend/ 100308/ 145-2C11/ 1:200
CD4/ PE-Texas red/ Invitrogen/ MCD0417/ RM4-5/ 1:200
CD4/ AF700/ Biolegend/ 100536/ RM4-5/ 1:200
CD4/ PE/ Biolegend/ 100512/ RM4-5/ 1:200
CD4/ BV711/ Biolegend/ 100550/ RM4-5/ 1:200
CD8a/ Pe-Cy7/ Biolegend/ 100722/ 53-6.7/ 1:200
CD8a/ BV785/ Biolegend/ 100750/ 53-6.7/ 1:200
CD11b/ BV605/ Biolegend/ 101257/ M1/70/ 1:200
CD11b/ PE/ Biolegend/ 101208/ M1/70/ 1:200
CD11b/ PerCP-Cy5.5/ eBioscience/ 45-0112-82/ M1/70/ 1:200
CD11b/ PeCy7/ Biolegend/ 101216/ M1/70/ 1:200
CD11c/ BV421/ Biolegend/ 117330/ N418/ 1:200
CD25/ AF488/ Invitrogen/ 53-0251-82/ PC61.5/ 1:200
CD25/ PE-Cy7/ Invitrogen/ 25-0251-82/ PC61.5/ 1:200
CD28/unconjugated/Biolegend/102112/37.51/1:1000
CD45/ BV650/ Biolegend/ 103151/ 30-F11/ 1:200
CD45/ AF700/ BD Biosciences/ 56-0451-82/ 30-F11/ 1:200
CD45/ BV510/ Biolegend/ 103137/ 30-F11/ 1:200
CD64/ FITC/ Biolegend/ 139316/ X54-5/7.1/ 1:200
CD64/ PE/ Biolegend/ 139304/ X54-5/7.1/ 1:200
CD127/ PE/ Biolegend/ 135010/ A7R34/ 1:200
CD90.2/ BV786/ Biolegend/ 105331/ 30-H12/ 1:200
CXCR4/ PE/ Biolegend/ 146506/ L276F12/ 1:200
CXCR4/ Biotin/ Biolegend/ 146516/ L276F12/ 1:100
F4/80/ BV786/ Biolegend/ 123141/ BM8/ 1:200
F4/80/ APC/ eBioscience/ 17-4801-82/ BM8/ 1:200
FoxP3/ BV421/ Invitrogen/ 48-5773-82/ FJK-16s/ 1:100
FoxP3/ FITC/ Invitrogen/ 11-5773-82/ FJK-16s/ 1:100
GITR/ APC/ Biolegend/ 126312/ DTA-1/ 1:200
IFNgamma/unconjugated/BioLegend/505834/XMG1.2/1:100
Ki67/ PE/ Invitrogen/ 12-5698-82/ SolA15/ 1:100
Ly6C/ AF700/ BD Bioscience/ 561237/ AL-21/ 1:200
Ly6C/ PerCP-Cy5.5/ BD Bioscience/ 560525/ AL-21/ 1:200
Ly6C/ PE-Cy7/ BD Bioscience/ 560593/ AL-21/ 1:200
Ly6G/ APC-Cy7/ Biolegend/ 127624/ 1A8/ 1:200
MHC II / FITC/ Biolegend/ 107606/ M5/114.15.2/ 1:200
MHC II/ BV650 / BD Bioscience/ 563415/ M5/114.15.2/ 1:200
NRP1/ FITC/ R&D Systems/ FAB5994G/ 761705/ 1:200
RORγt/ PE/ eBioscience/ 12-6981-82/ B2D/ 1:100
TBET/ APC/ Invitrogen/ 17-5825-82/ 4B10/ 1:100
TCRβ/ BV711/ Biolegend/ 109243/ H57-597/ 1:200
IL1β pro-form/ Pe-Cy7/ Miltenyi Biotec/ 130-109-043/ REA577/ 1:10
IL10/ BV605/ BD Biosciences/ 564082/ JES5-16E3/ 1:100

Validation

Antibody/ Colour/ Supplier/ Validation
B220/ APC-Cy7 / Invitrogen/ https://www.thermofisher.com/antibody/product/CD45R-Antibody-clone-RA3-6B2-Monoclonal/A15399
CD3ε/unconjugated/Biolegend/https://www.biolegend.com/en-us/products/purified-anti-mouse-cd3epsilon-antibody-28
CD3ε/ PerCP-Cy5.5 / eBioscience/ https://www.thermofisher.com/antibody/product/CD3e-Antibody-clone-145-2C11-Monoclonal/45-0031-82
CD3ε/ BV711 / Biolegend/ https://www.biolegend.com/en-us/products/brilliant-violet-711-anti-mouse-cd3epsilon-antibody-11975
CD3ε/ PE-Cy7 / Biolegend/ https://www.biolegend.com/en-us/products/pe-cy7-anti-mouse-cd3epsilon-antibody-1899
CD3ε/ PE / Biolegend/ https://www.biolegend.com/en-us/products/pe-anti-mouse-cd3epsilon-antibody-25
CD4/ PE-Texas red / Invitrogen/ https://www.thermofisher.com/antibody/product/CD4-Antibody-clone-RM4-5-Monoclonal/MCD0417
CD4/ AF700 / Biolegend/ https://www.biolegend.com/en-us/products/alexa-fluor-700-anti-mouse-cd4-antibody-3386
CD4/ PE / Biolegend/ https://www.biolegend.com/en-us/products/pe-anti-mouse-cd4-antibody-482
CD4/ BV711 / Biolegend/ https://www.biolegend.com/en-us/products/brilliant-violet-711-anti-mouse-cd4-antibody-7925
CD8a/ Pe-Cy7 / Biolegend/ https://www.biolegend.com/en-us/products/pe-cy7-anti-mouse-cd8a-antibody-1906
CD8a/ BV785 / Biolegend/ https://www.biolegend.com/en-us/products/brilliant-violet-785-anti-mouse-cd8a-antibody-7957
CD11b/ BV605 / Biolegend/ https://www.biolegend.com/en-us/products/brilliant-violet-605-anti-mouse-human-cd11b-antibody-7637
CD11b/ PE / Biolegend/ https://www.biolegend.com/en-us/products/pe-anti-mouse-human-cd11b-antibody-349
CD11b/ PerCP-Cy5.5 / eBioscience/ https://www.thermofisher.com/antibody/product/CD11b-Antibody-clone-M1-70-Monoclonal/45-0112-82

CD11b/ PeCy7 / Biolegend/ https://www.biolegend.com/en-us/products/pe-cy7-anti-mouse-human-cd11b-antibody-1921
CD11c/ BV421 / Biolegend/ https://www.biolegend.com/en-us/products/brilliant-violet-421-anti-mouse-cd11c-antibody-7149
CD25/ AF488 / Invitrogen/ https://www.thermofisher.com/antibody/product/CD25-Antibody-clone-PC61-5-Monoclonal/53-0251-82
CD25/ PE-Cy7 / Invitrogen/ https://www.thermofisher.com/antibody/product/CD25-Antibody-clone-PC61-5-Monoclonal/25-0251-82
CD28/unconjugated/Biolegend/https://www.biolegend.com/en-us/products/purified-anti-mouse-cd28-antibody-117
CD45/ BV650 / Biolegend/ https://www.biolegend.com/en-us/products/brilliant-violet-650-anti-mouse-cd45-antibody-11987
CD45/ AF700 / BD Biosciences/ https://www.thermofisher.com/antibody/product/CD45-Antibody-clone-30-F11-Monoclonal/56-0451-82
CD45/ BV510 / Biolegend/ https://www.biolegend.com/en-us/products/brilliant-violet-510-anti-mouse-cd45-antibody-7995
CD64/ FITC / Biolegend/ https://www.biolegend.com/en-us/products/fitc-anti-mouse-cd64-fcgammari-antibody-12422
CD64/ PE / Biolegend/ https://www.biolegend.com/en-us/products/pe-anti-mouse-cd64-fcgammari-antibody-6691
CD127/ PE / Biolegend/ https://www.biolegend.com/en-us/products/pe-anti-mouse-cd127-il-7ralpha-antibody-6190
CD90.2/ BV786 / Biolegend/ https://www.biolegend.com/en-us/products/brilliant-violet-785-anti-mouse-cd90-2-antibody-9106
CXCR4/ PE / Biolegend/ https://www.biolegend.com/en-us/products/pe-anti-mouse-cd184-cxcr4-antibody-9057
CXCR4/ Biotin / Biolegend/ https://www.biolegend.com/en-us/products/biotin-anti-mouse-cd184-cxcr4-antibody-14104
F4/80/ BV786 / Biolegend/ https://www.biolegend.com/en-us/products/brilliant-violet-785-anti-mouse-f4-80-antibody-9919
F4/80/ APC / eBioscience/ https://www.thermofisher.com/antibody/product/F4-80-Antibody-clone-BM8-Monoclonal/17-4801-82
FoxP3/ BV421 / Invitrogen/ https://www.thermofisher.com/antibody/product/FOXP3-Antibody-clone-FJK-16s-Monoclonal/48-5773-82
FoxP3/ FITC / Invitrogen/ https://www.thermofisher.com/antibody/product/FOXP3-Antibody-clone-FJK-16s-Monoclonal/11-5773-82
GITR/ APC / Biolegend/ https://www.biolegend.com/en-us/products/apc-anti-mouse-cd357-gitr-antibody-4646
IFNgamma/unconjugated/BioLegend/https://www.biolegend.com/en-us/products/ultra-leaf-purified-anti-mouse-ifn-gamma-antibody-7752
Ki67/ PE / Invitrogen/ https://www.thermofisher.com/antibody/product/Ki-67-Antibody-clone-SolA15-Monoclonal/12-5698-82
Ly6C/ AF700 / BD Bioscience/ https://www.bdbiosciences.com/us/reagents/research/antibodies-buffers/immunology-reagents/anti-mouse-antibodies/cell-surface-antigens/alexa-fluor-700-rat-anti-mouse-ly-6c-al-21/p/561237
Ly6C/ PerCP-Cy5.5 / BD Bioscience/ https://www.bdbiosciences.com/us/reagents/research/antibodies-buffers/immunology-reagents/anti-mouse-antibodies/cell-surface-antigens/percp-cy55-rat-anti-mouse-ly-6c-al-21/p/560525
Ly6C/ PE-Cy7 / BD Bioscience/ https://www.bdbiosciences.com/eu/reagents/research/antibodies-buffers/immunology-reagents/anti-mouse-antibodies/cell-surface-antigens/pe-cy7-rat-anti-mouse-ly-6c-al-21/p/560593
Ly6G/ APC-Cy7 / Biolegend/ https://www.biolegend.com/en-us/products/apc-cy7-anti-mouse-ly-6g-antibody-6755
MHC II / FITC / Biolegend/ https://www.biolegend.com/en-us/products/fitc-anti-mouse-i-a-i-e-antibody-366
MHC II/ BV650 / BD Bioscience/ https://www.bdbiosciences.com/us/reagents/research/antibodies-buffers/immunology-reagents/anti-mouse-antibodies/cell-surface-antigens/bv650-rat-anti-mouse-i-ai-e-m5114152-also-known-as-m5114/p/563415
NRP1/ FITC / R&D Systems/ https://www.rndsystems.com/products/mouse-neuropilin-1-alexa-fluor-488-conjugated-antibody-761705_fab5994g
RORγ(t)/ PE / eBioscience/ https://www.thermofisher.com/antibody/product/ROR-gamma-t-Antibody-clone-B2D-Monoclonal/12-6981-82
TBET/ APC / Invitrogen/ https://www.thermofisher.com/antibody/product/T-bet-Antibody-clone-eBio4B10-4B10-Monoclonal/17-5825-82
TCRβ/ BV711 / Biolegend/ https://www.biolegend.com/en-us/products/brilliant-violet-711-anti-mouse-tcr-beta-chain-antibody-13539
IL1β pro-form/ Pe-Cy7 / Miltenyi Biotec/ https://www.miltenyibiotec.com/CA-en/products/macs-flow-cytometry/antibodies/primary-antibodies/anti-il-1b-pro-form-antibodies-mouse-rea577-1-10.html#pe-vio770:30-ug-in-1-ml
IL10/ BV605 / BD Biosciences/ https://www.bdbiosciences.com/us/applications/research/t-cell-immunology/th-2-cells/intracellular-markers/cytokines-and-chemokines/mouse/bv605-rat-anti-mouse-il-10-jes5-16e3/p/564082

## Animals and other organisms

Policy information about studies involving animals; ARRIVE guidelines recommended for reporting animal research

| | |
|---|---|
| Laboratory animals | The following mouse strains were used:<br>DBA/1 males age 9-10 weeks at collagen immunisation<br>DEREG males age 8-15 weeks at collagen immunisation<br>CD4-Bmal-/- Per2::luc males, age 8-20 weeks |
| Wild animals | The study did not involve wild animals |
| Field-collected samples | The study did not involve samples collected in the field |
| Ethics oversight | Local ethical approval was provided by the University of Manchester Animal Welfare Ethical Review Board (AWERB) |

Note that full information on the approval of the study protocol must also be provided in the manuscript.

## Flow Cytometry

### Plots

Confirm that:

☒ The axis labels state the marker and fluorochrome used (e.g. CD4-FITC).

☒ The axis scales are clearly visible. Include numbers along axes only for bottom left plot of group (a 'group' is an analysis of identical markers).

☒ All plots are contour plots with outliers or pseudocolor plots.

☒ A numerical value for number of cells or percentage (with statistics) is provided.

### Methodology

| | |
|---|---|
| Sample preparation | Cells were digested from limbs using collagenase from Clostridium histolyticum with a digestion time of 45 mins at 37°C. Cells were extracted from lymph nodes and spleen using mechanical disruption. Cell staining was carried out in a 96 well V bottomed plate. After live/dead staining (LIVE/DEAD fixable blue dead stain kit, ThermoFisher Scientific) and blocking (1:100 anti mouse CD16/CD32, Fisher Scientific, UK) cells were stained with a panel of antibodies. For intracellular staining an eBioscience FoxP3/ Transcription factor staining kit (Thermo Fisher) was utilized as per kit instructions. Where intracellular cytokine expression was analysed, isolated joint cells were first incubated in media containing monensin (1:1000, BioLegend UK) for 3.5h at 37°C before staining commenced. After application of antibodies targeting extracellular proteins, cells were incubated with a fixation/ permeabilisation solution (FoxP3/Transcription factor staining kit, Thermo Fisher) for 45min before intracellular staining. Amine Reactive Compensation beads (Thermo Fisher Scientific) were utilized with the LIVE/DEAD stain for compensation controls. One Comp eBeads (Thermo Fisher Scientific) were utilized to produce single stained compensation controls. |
| Instrument | Cells were analysed on an LSR II or BD Biosciences LSR Fortessa<br>Fluorescence assisted cell sorting was undertaken using either a BD Biosciences FACS Aria Fusion or BD Influx (BD Biosciences). |
| Software | BD FACS Diva was utilised to collect FACS data and data was analysed using Flow Jo. |
| Cell population abundance | Details of cell population abundance are provided in the manuscript |
| Gating strategy | Gating strategies are shown in the manuscript. For each experiment cells were first gated on FSC(A) and SSC(A) to exclude debris. FSC(H) and FSC(A) was utilised to identify single cells and subsequently cells were gated for Live/dead to exclude dead cells. Gating strategies are shown in the manuscript and distinct cell populations were gated as follows:<br>MHC II low macrophages: Single/live/CD45+/CD11b+/Ly6C+/F4/80+/MHCII low<br>MHC II high macrophages: Single/live/CD45+/CD11b+/Ly6C+/F4/80+/MHCII high<br>Neutrophils: Single/live/CD45+/CD11b+/Ly6C+/F4/80-/Ly6G+<br>CD3e T cells: Single/live/CD45+/B220-/CD3e+<br>CD4+ T cells: Single/live/CD45+/B220-/CD3e+/CD4+<br>CD8+ T cells:Single/live/CD45+/B220-/CD3e+/CD8+<br>Th1 cells: Single/live/CD45+/B220-/CD3e+/CD4+/Tbet+<br>Th17 cells: Single/live/CD45+/B220-/CD3e+/CD4+/RORgammaT+<br>Treg (using intracellular markers): Single/live/CD45+/B220-/CD3e+/CD4+/CD25+/FoxP3+<br>Treg (using only extracellular markers): Single/live/CD45+/CD3e+/CD4+/CD25+/CD127-<br>Ly6C hi monocytes: Single/live/CD45+/CD11b+/Ly6G-/F4/80-/Ly6C hi<br>Ly6C low monocytes: Single/live/CD45+/CD11b+/Ly6G-/F4/80-/Ly6C low<br>Dendritic cells: Single/live/CD45+/TCRbeta-/CD11c+/CD11b+/CD64-/CD4- |

☒ Tick this box to confirm that a figure exemplifying the gating strategy is provided in the Supplementary Information.
